# Supplementary material for: A Critical View of the Application of the APEX Software (Aqueous Photochemistry of Environmentally-Occurring Xenobiotics) to Predict Photoreaction Kinetics in Surface Freshwaters
Source: Molecules. 2019 Dec 18;25(1):9. doi: 10.3390/molecules25010009 (PMC7017383; doi:10.3390/molecules25010009)
Supplement: Supplementary file 1 [file molecules-25-00009-s001.zip › 3.2.4_gcc-4.4.0.zip!/3.2.4_gcc-4.4.0/3.2.4_gcc-4.4.0/mingw32/doc/a2dll/a2dll.html]

a2dll: An utility (to help) to convert static library into Win32 DLL


# a2dll: An utility (to help) to convert static library into Win32 DLL

## Synopsis

a2dll is shell script (see requirements) to
automotize process of converting existing static libraries (produced
by gnu-win32 tools, of course) into DLL. First of all, yes it's possible:
if you have binary static distribution of some library (i.e. library
itself and its headers), that's all you need to convert it to DLL and
use in your programs. Read HOWTO for
underlying magic. So, you may not waste time if you need DLL: just
grab existing static distribution and convert. Also, you may use it to
build Win32 DLL of your library. Also, until GNU libtool will allow
seamless building of Win32 DLLs, you may build static lib (what
libtool of course supports) and then convert it to DLL.
> a2dll <static\_lib> [-o <dll\_name>] [<linker\_flags>] [--relink]

where:

<static\_lib>
:   Static library you want to convert

-o <dll\_name>
:   Name of resulting dll. If not given, three first chars of input
    name are stripped and .a suffix replaced with .dll .
    So, from 'libfoo.a' you'll get 'foo.dll'.

<linker\_flags>
:   Linker flags:

    - Use '-s' to get library without debugging symbols and information.- Use '--driver-name=<name>' to link library with specified
        linker (well, compiler, to be precise). For example, for C++ library use
        --driver-name=g++ .- You should list all libraries on which your library depends with
          -l switches and directories they are reside in with -L
          switches. For example, if your library uses PCRE library you just built and
          not yet installed, use something like -L../pcre -lpcre

--relink
:   Skip exploding library stage (see below). Use this flag to continue
    process after some error occured.

## Performing

a2dll works in following way:

1. If you did not specify --relink option,
   explodes source library under newly-created .dll subdirectory.- Links all resulting objects into DLL with exporting all non-static
     symbols. On this stage, link errors
     (such as underfined symbols) may occur. In such case, a2dll terminates
     and all linker messages are available in 'ld.err' file. You
     should correct errors (mostly by finding out additional
     dependecies, but sometimes by deleting 'superfluos' objects under .dll)
     and re-run a2dll with all the options you gave it before,
     plus new dependencies, plus --relink flag. You may need to
     repeat this several times.- Renames original static library with suffix .static .- Creates import library for produced DLL with the name of original
         static library.- Check whether DLL exports data symbols. If no, congratulations,
           you've done. However, if some present, it lists all of them in file
           '<dll\_name>.data' . Presense of such symbols generally
           means that you should patch library's headers to mark those symbols
           as dll-imported. But don't hurry with that, first, do following:
           1. Look into <dll\_name>.data file. If all what you see
              is something like 'internal\_counter\_of\_bogons' or
              '\_ksdauso', don't worry - those symbols are hardly part of
              external interface of the library.- If all you need is to link your application against that
                library, try it. If it succeeds, congratulation.- Only if above is failed, or you are going to distribute produced
                  library, so you need to be sure that everything is ok, proceed with
                  marking symbols in headers. Read Static2DLL
                  HOWTO for more information on suggested ways of doing this. Use
                  'grep -f <dll\_name>.data \*.h' command to find
                  out where offending symbols defined in library headers.

## Examples

Since converting static libraries to DLLs is not fully automated and
formal process, some experience with it is required. Learing
by example is known to be one of the efficient way of communicating
experince, so I would like to provide some realistic examples of
converting statics to DLLs with the help of a2dll.

### Zlib

Build libz.a . Now, run 'a2dll libz.a'. It builds cleanly,
but warns us about data symbols. Let's look at them:
> ```
> inflate_mask
> z_errmsg
> ```

What they could be. The first one is probably some internal variable,
while second is probably array of error messages. As we know, zlib
provides functional way of getting error messages, something like. So
our hypothesis is that job's done. Let's prove it:
'grep -f z.dll.data zlib.h'. Yes, we're right: no mentioning
of those symbols in interface header file.

### libstdc++

I've got an idea to DLLize libstdc++ coming with my mingw32 distribution.
'a2dll "libstdc++.a"'. Note that we don't use
--driver-name=g++ - that option need to be used when we link
something *against* libstdc++ . But when we link libstdc++
*itself*, we need libc (whatever it is in mingw32), nothing else.
But, process aborts due to linker errors. ld.err tells us:
> ```
> strerror.o(.text+0x303): undefined reference to `sys_nerr'
> vfork.o(.text+0x7): undefined reference to `fork'
> waitpid.o(.text+0x15): undefined reference to `wait'
> ```

Well, strerror, vfork, waitpid are libc functions, what they do in
libstdc++? Probably, stubs, delete them and
'a2dll "libstdc++.a" --relink'. Of course,
stdc++.dll.data is here. Looking into it, I may tell you
that everything starting with '\_\_ti<digit>' is RTTI
internal data structures and everything starting with
'\_vt$' is virtual tables (use c++filt if in doubt),
you can leave them alone.
(If so, why I don't filter them? Because "you can leave them alone"
is hypothesis for now, I haven't linked too much C++ libraries to
be sure). From the rest, there's stuff starting
with '\_IO\_'. That's probably some internal variables, let's
don't do anything about them, unless we'll be forced to. Than, as
c++filt shows, there're some static members of templated classes. Darkness.
Forget for now. Than, there's 'io\_defs\_\_'. Does your C++ application
reference something like that? Mine not. So, what is left? Our four
happy friends, cin, cout, cerr, and clog. Do mark them as
\_\_declspec(dllimport) in iostream.h.

### Some C++ library

Suppose we have following file:

```
#include <iostream.h>

void foo()
{
  cout<<"hi!"<<endl;
}
```

and want to turn it into DLL. Create static liba.a from it. Now,
'a2dll liba.a --driver-name=g++'. Well, our DLL contains
single function, why then it complains about data symbols? Oh, it's
those stupid RTTI structures. Next time, compile with -fno-rtti unless
you really need it, ok? Ditto for -fno-exceptions .

## Requirements

a2dll requires POSIX shell (sh) to run. It is developed and
tested with ash from
PW32 distribution. Additionally,
a2dll requires following utilities to perform its tasks:

- GNU fileutils: mkdir, mv, rm- GNU textutils: wc- GNU grep- GNU awk- GNU binutils: dllwrap, dlltool (and the rest of binutils and gcc, of course)- pexports, an utility to dump symbols exported by dll. You'll need a
            version 0.43 or above, capable of distinguishing between code and data symbols, as one
            from here.

---

*Paul Sokolovsky*
